# The role of ultrasound-guided perineural injection of the tibial nerve with a sub-anesthetic dosage of lidocaine for the diagnosis of tarsal tunnel syndrome

**DOI:** 10.3389/fneur.2023.1135379

**Published:** 2023-04-17

**Authors:** Álvaro Iborra, Manuel Villanueva, Stephen L. Barrett, Lorena Vega-Zelaya

**Affiliations:** ^1^Unit for Ultrasound-Guided Surgery, Hospital Beata María Ana, Madrid, Spain; ^2^Avanfi Institute, Madrid, Spain; ^3^Department of Podiatry, School of Health Sciences, University of La Salle, Madrid, Spain; ^4^US Neuropathy Centers, Atlanta, GA, United States; ^5^Clinical Neurophysiology, Hospital Universitario de La Princesa, Madrid, Spain

**Keywords:** near-nerve needle sensory technique, tibial nerve, nerve conduction studies, neurophysiology, nerve functional capacity, tarsal tunnel syndrome, nerve entrapment

## Abstract

**Background:**

Tarsal tunnel syndrome (TTS) involves entrapment of the tibial nerve at the medial ankle beneath the flexor retinaculum and its branches, the medial and lateral plantar nerves, as they course through the porta pedis formed by the deep fascia of the abductor hallucis muscle. TTS is likely underdiagnosed, because diagnosis is based on clinical evaluation and history of present illness. The ultrasound-guided lidocaine infiltration test (USLIT) is a simple approach that may aid in the diagnosis of TTS and predict the response to neurolysis of the tibial nerve and its branches. Traditional electrophysiological testing cannot confirm the diagnosis and only adds to other findings.

**Methods:**

We performed a prospective study of 61 patients (23 men and 38 women) with a mean age of 51 (29–78) years who were diagnosed with idiopathic TTS using the ultrasound guided near-nerve needle sensory technique (USG-NNNS). Patients subsequently underwent USLIT of the tibial nerve to assess the effect on pain reduction and neurophysiological changes.

**Results:**

USLIT led to an improvement in symptoms and nerve conduction velocity. The objective improvement in nerve conduction velocity can be used to document the pre-operative functional capacity of the nerve. USLIT may also be used as a possible quantitative indicator of whether the nerve has the potential to improve in neurophysiological terms and ultimately inform prognosis after surgical decompression.

**Conclusion:**

USLIT is a simple technique with potential predictive value that can help the clinician to confirm the diagnosis of TTS before surgical decompression.

## 1. Introduction

Tarsal tunnel syndrome (TTS) is a common peripheral mononeuropathy that is not readily acknowledged by treating physicians. It is believed to be caused by entrapment of the tibial nerve beneath the flexor retinaculum in the medial ankle and its two distal branches, the medial and lateral plantar nerves, by the deep fascia of the abductor hallucis muscle.

The clinical course of TTS varies widely from patient to patient but often starts with pain in the heel and/or foot, with burning sensation, toe numbness, loss of plantar sensation, heaviness or tightness in the sole of the foot, intolerance of shoe pressure, and nocturnal symptoms. The symptoms are often exacerbated by increased activity (1, 2). One of the main problems when treating TTS is the difficulty in making an accurate diagnosis. There is currently no universal reliable test for diagnosing TTS, and diagnosis usually involves a combination of the clinical history, imaging tests, and electromyography (EMG), which is often unreliable (3–5). The ultrasound guided near-nerve needle sensory technique (USG-NNNS) has proven to be highly effective for reaching a definitive diagnosis in TTS, although it is not widely available in clinical practice (6). Improvements in ultrasound technology in the form of high-frequency probes and modern devices enable us to differentiate between the histological components of the peripheral nerve, such as the hypoechoic fascicles and the hyperechoic epifascicular epineurium. We can even perform semiquantitative measurements of the peripheral nerve, in both the acute phase and the chronic phase of neuropathy, thereby improving ultrasound-guided invasive techniques for diagnosis (7–9).

Physical examination is based on findings such as a positive provocation sign, a positive Hoffman-Tinel sign, and the forced foot eversion and flexion maneuver (10–14). While the Hoffman-Tinel test may be positive in more than half of patients with this condition, it is often negative in late stages of neural degeneration. Constant pressure on the nerve may produce radiation of pain with tingling and numbness, which is known as the Valleix phenomenon (14–18). The measurement of pressure within the tarsal tunnel can help in the diagnosis of the condition (19), although this type of testing is difficult both for the physician and for the patient and is only minimally implemented in teaching hospitals.

Diagnostic local anesthetic blocks, specifically with lidocaine, are well established among clinicians and widely used to determine the location of the pain generator and to predict whether neuropathic pain decreased after conservative or surgical treatment (20–24). However, there is scant objective scientific evidence for performance of diagnostic peripheral nerve block before surgery.

To our knowledge, only 4 studies have assessed the predictive value of diagnostic nerve block with local anesthesia for pain relief as a positive outcome after surgery, and the results differ considerably. Both Stokvis et al. and Malessy et al. found that lidocaine was not a reliable predictor of the outcome of surgical treatment. Stokvis et al. did not obtain a positive result for lidocaine as a predictor of positive outcomes in surgery. However, those who did not respond to the nerve block were not operated on, thereby introducing a selection bias (23, 25). Similarly, Malessy et al. found that the predictive ability of lidocaine block was poor. Therefore, the authors recommended careful interpretation of responses to the block when selecting patients for surgery, as these may not be as predictive as generally presumed (25).

Barrett et al., on the other hand, found that when lidocaine was injected at subanesthetic doses adjacent to the common peroneal nerve under high-resolution sonography, pain often improved and the motor strength of the anterior muscle group (tibialis anterior, extensor digitorum longus, and extensor hallucis longus) was routinely evidenced by increased dorsiflexion of the foot due to improved muscle function. These findings were not present prior to the administration of the ultrasound-guided infiltration. The authors named this phenomenon, the “Phoenix Sign,” since the common peroneal nerve that previously did not function “rose from the ashes” like the mythical Greek bird, and the patients' drop foot resolved temporarily (26, 27).

Unlike Barrett et al., Nirenberg (28) obtained a similar result after injecting lidocaine into the long peroneal muscle (through which the common peroneal nerve runs) as a test and to predict decompression of the common peroneal nerve.

Given the contradictory findings reported in the scant available literature, our study combined quantitative and neurophysiological assessment with subanesthetic doses of lidocaine. This objective approach involved infiltrating a subanesthetic dose of a local anesthetic next to the tibial nerve using USG-NNNS. After infiltration, we observe not only a clinical improvement in the form of reduced neuropathic pain expressed subjectively by the patient, but also an objective improvement in nerve conduction velocities.

## 2. Materials and methods

A prospective study was carried out on 61 patients (23 men and 38 women; mean age, 51 years) who had experienced signs and symptoms of TTS and had compatible electromyogram/nerve conduction study (EMG/NCS) findings indicating entrapment of the tibial nerve and/or its unilateral or bilateral distal branches. The neurophysiologist carried out a complete protocol-based assessment of the peripheral nerves in the lower limbs to rule out other nerve disorders. Patients with a nerve disorder other than TTS were excluded.

All patients with signs and symptoms of TTS and a positive EMG/NCS result without radiculopathy or degenerative nerve disease were included and underwent USLIT.

USLIT was performed on the tibial nerve with 1% lidocaine 0.5 ml, and the change in neuropathic symptoms was recorded 5 min later.

**Figure 1:**
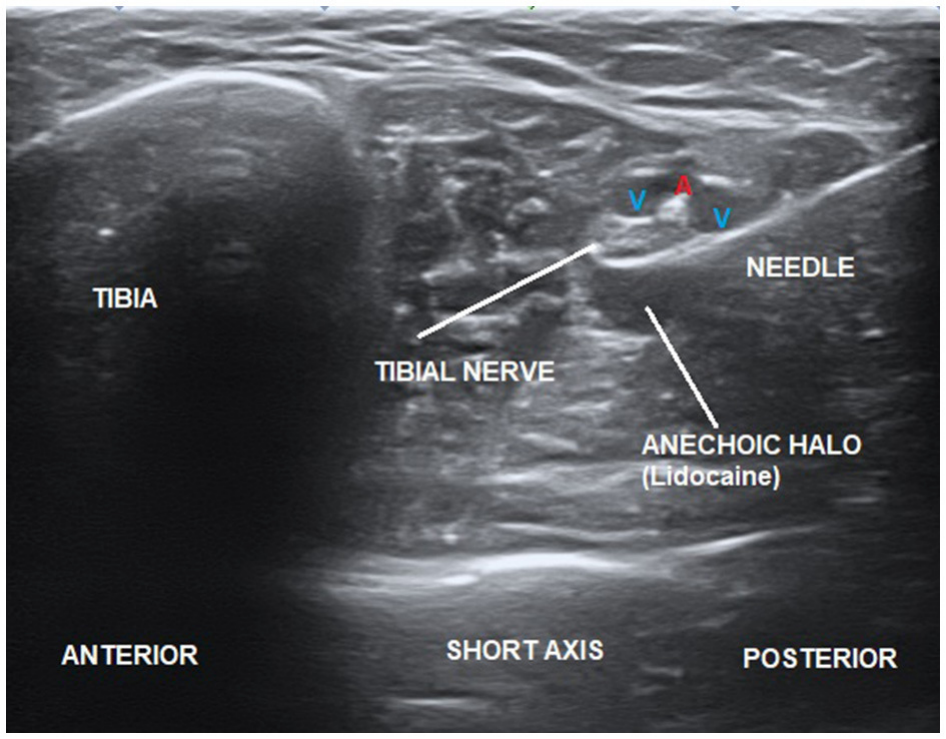
Ultrasound-guided injection of lidocaine close to the tibial nerve producing an anechoic halo.

The injection was administered with a 22-gauge needle under real-time dynamic ultrasound guidance. Lidocaine was injected close to the tibial nerve, thus producing an anechoic halo surrounding the epifascicular epineurium. The approach for the ultrasound-guided injection was made on a short axis to the tibial nerve using the in-plane technique in a posterior-to-anterior direction (Alpinion ECube15) with an 8- to 17-MHz linear transducer and the Needle Vision Plus™ software package (Alpinion Medical Systems, Bothell, WA, USA) (Figure 1).

**Figure 2:**
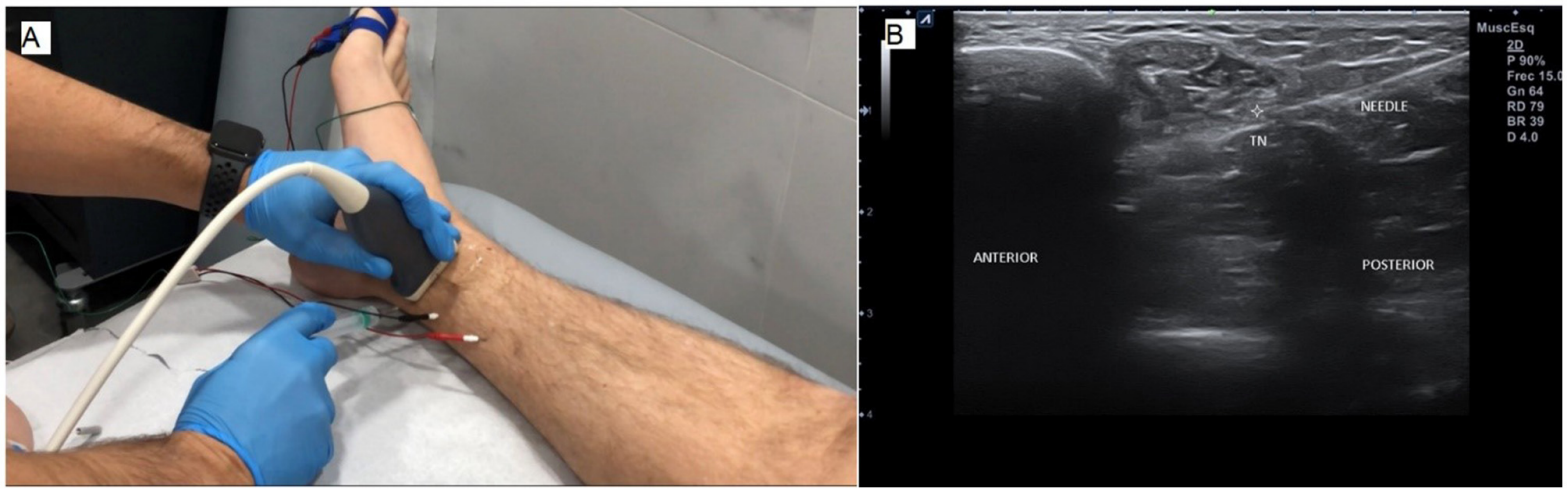
**(A)** Ultrasound-guided lidocaine infiltration test (USLIT) and the active and reference needle position for recording is displayed. **(B)** Ultrasound image showing the position of the needle for infiltration with respect to the tibial nerve (TN).

A minimum 24-h interval was left after USLIT to monitor possible delayed effects of lidocaine. All patients then underwent a study with USG-NNNS, according to the described protocol. The active needle recording electrode was inserted and guided using ultrasound close to the tibial nerve in the ankle above the flexor retinaculum. The reference needle electrode was placed subcutaneously at the same level as the active electrode at 3–4 cm (Figure 2A). The adequacy of the needle position was confirmed by stimulating the nerve through the active electrode. The position was considered correct when the toes contracted minimally with <1 mA for a stimulus of 50 μs in duration. Digits I and V were then stimulated separately (6).

Those who reported positive sensory findings for the tibial nerve and/or its distal branches received (without removing the USG-NNNS needle) (6) 0.5 ml of 1% lidocaine adjacent to the tibial nerve, immediately distal to the recording needle (Figure 2A).

Five minutes after infiltration, neurophysiological testing was performed again, and changes in conduction velocity were recorded (Figure 2B).

The changes recorded before and after application of USLIT included neurophysiological changes, improvement/no improvement in symptoms, and changes in nerve conduction velocity with USG-NNNS. These were analyzed to identify statistically significant relationships.

## 3. Results

No improvement in neuropathic symptoms was observed in 17 of the 61 patients; an improvement was observed in the remaining 44. The results showed that conduction velocity also improved in patients whose neuropathic symptoms improved with USLIT. Patients with no improvement in clinical symptoms with USLIT did not experience an increase in sensory conduction velocity with USLIT.

In the 61 patients whose symptoms improved with USLIT, 3 had sensorimotor neuropathy of the tibial nerve. Sensory conduction velocity improved in all 3 cases after lidocaine injection, although only 1 also experienced improved motor conduction (6).

**Figure 3:**
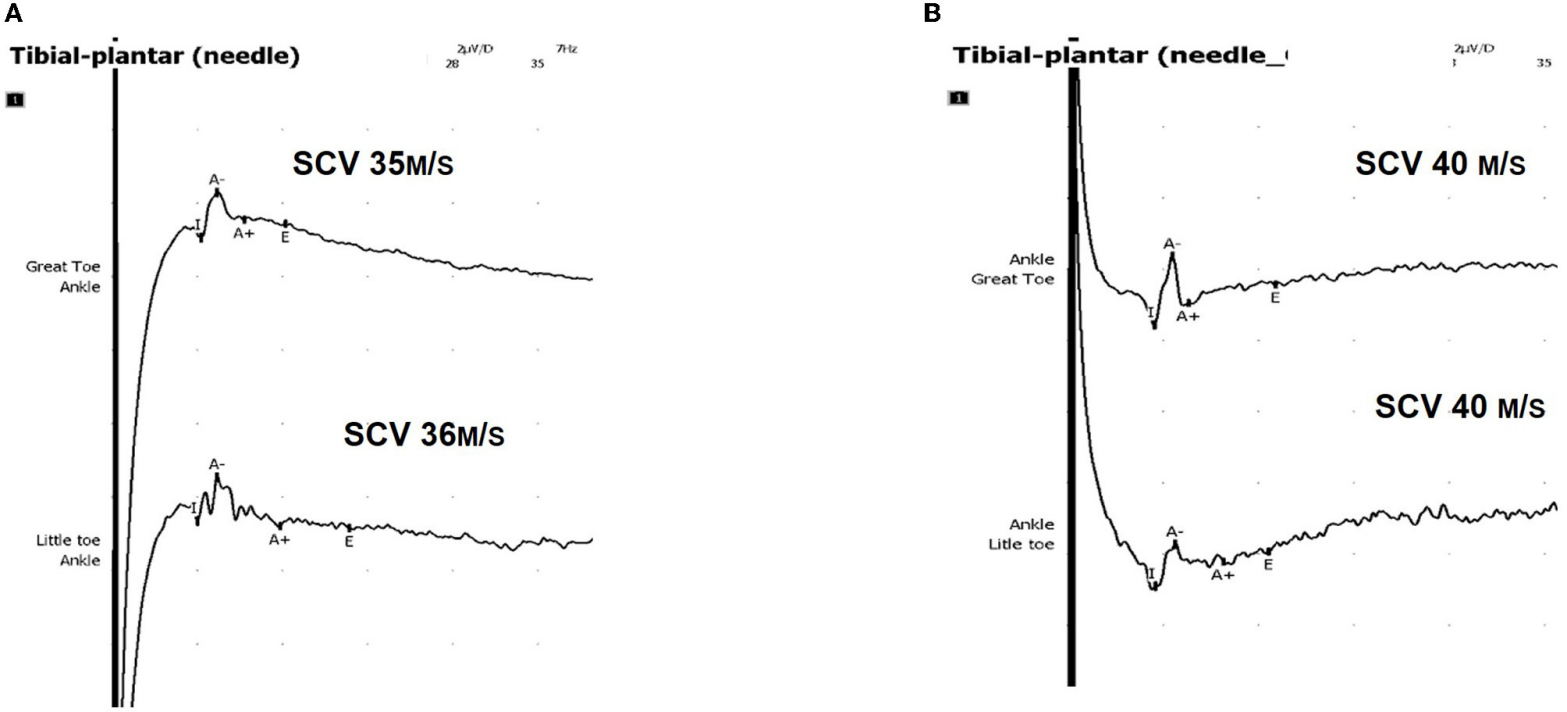
Ultrasound-guided near-nerve needle sensory technique (USG-NNNS) applied to the tibial nerve. **(A)** Baseline recording, pre-USLIT. **(B)** Recording post-USLIT shows improvement with increased sensory conduction velocity (SCV).

Therefore, after USLIT, a significant correlation was found between clinical improvement and increased sensory conduction velocity in 72.1% of patients. The clinical improvement in neuropathic symptoms with USLIT was transient, lasting ~60 min on average (Figure 3).

## 4. Discussion

As with other compressive neuropathies, TTS often produces neuropathic pain, a burning sensation, paresthesia, heaviness or tightness in the sole of the foot, shoe pressure intolerance, and nocturnal symptoms (1).

The diagnosis of entrapment of the tibial nerve and its branches, as with that of other compressive neuropathies, is based mainly on the patient's clinical history and physical examination, which may include provocative signs (e.g., the Valleix and Tinel signs), or on nerve blocks, EMG/NCS, ultrasound, and magnetic resonance imaging (26, 29, 30).

The objective of our study was to provide neurophysiological evidence for diagnostic peripheral nerve blocks with lidocaine so that USLIT can become more established and widespread and thus enable physicians to more accurately diagnose TTS, determine the precise location of the nerve compression, and, possibly, predict whether surgical treatment would improve the patient's neuropathic symptoms.

Literature supporting this widespread practice is scant and contradictory. Stokvis et al. did not obtain a positive result for lidocaine as a positive predictor of outcomes in their surgical procedure. However, patients who did not respond to preoperative block were not operated on, thereby introducing considerable selection bias (23, 25).

Similarly, Malessy et al. found that the predictive ability of lidocaine block was poor. Therefore, the authors recommended careful interpretation of responses to the block when selecting patients for surgery, as these may not be as predictive as generally presumed (25).

In contrast, Barrett et al. (27) and Nirenberg (28) obtained satisfactory and predictive results with lidocaine in surgical treatment, postulating that the patients who responded to the test with lidocaine improved with surgery.

Barrett et al. (27) defined the positive “Phoenix Sign,” i.e., an increase in the motor force of the extensor hallucis longus after ultrasound-guided common peroneal nerve block with a dose of <0.5 ml and waiting 3–5 min to confirm clinical improvement.

In the present study, we followed the dose and latency of lidocaine reported by Barrett et al. More precisely, we used an ultrasound-guided perineural injection of 1% lidocaine 0.5 ml over the tibial nerve and waited 5 min to register the response in USG-NNNS.

The difference between our study and that of Barret et al. was that we evaluated the objective neurophysiological response of the tibial nerve after the nerve block, correlating it with an improvement in symptoms in patients previously diagnosed with TTS using USG-NNNS.

Clinical improvement and prediction with the lidocaine test are based on two hypotheses. The first is that of Barrett et al., in which the vasodilator effect of lidocaine enables a temporary increase in the local vascular supply at the site of nerve entrapment. Based on these findings, it could be concluded that peripheral nerve block can also improve the prognosis of both motor and sensory recovery after surgical nerve decompression (26).

Barrett et al. (27) further support the hypothesis that the effect is the result of vasodilation, as they showed in several patients whose motor strength improved with plain lidocaine; however, when these same patients were infiltrated with lidocaine and 1:200,000 epinephrine (vasoconstrictor), the authors did not observe the Phoenix Sign.

Using high-sensitivity Doppler, Ricci et al. evaluated the microcirculation of the peripheral nerve in both the acute and the chronic phase of neuropathy. This ultrasound study confirmed the hypothesis of the vasodilator effect of intraneural lidocaine. Further studies may focus on the use of Doppler imaging to visualize changes after lidocaine injection.

The second hypothesis is that proposed by Nirenberg (28), who reports that local anesthetic paralyzes the long lateral peroneal muscle, thus decompressing the nerve and improving the function and symptoms corresponding to the common peroneal nerve.

Our objective findings confirm that lidocaine at sub-anesthetic doses has a neurophysiological effect on the nerve, leading to improved conduction velocity. This is most likely an intraneural or perineural vasodilator effect that is not related to the relaxation of the muscle (27, 28).

Interestingly, we found that injecting lidocaine adjacent to the tibial nerve improves conduction velocity (if previously abnormal). This is also observed in the distal branches (medial and/or lateral plantar nerve).

Therefore, we conclude that performing USLIT on the tibial nerve will act on the medial and lateral plantar nerves without the need to perform the procedure directly on these distal branches, thus facilitating clinical application.

The association between the lidocaine test and the postoperative prognosis reported by Barrett et al. and Nirenberg et al. could result from a vasodilator effect of the drug when injected at sub-anesthetic doses. Since focal nerve entrapments create focal ischemia, this effect could lead to improved conduction velocity.

Therefore, the nerve still has the capacity to function when focal blood flow is improved. A positive “Phoenix Sign,” as described by Barrett et al., was observed in those patients who regained motor function of the anterior compartment in the form of the ability to dorsiflex the foot, which they did not possess before the block.

Barrett et al. and Nirenberg report motor improvement after the injection of lidocaine. In our study, 3 of the 61 patients had sensorimotor neuropathy of the tibial nerve. Sensory conduction velocity improved in all 3 cases after the injection of lidocaine, although only 1 patient also experienced an improvement in motor conduction. Moreover, we found that sensory neuropathy was much more common than motor impairment.

Our data enable us to speculate that studies relying on classic electromyography are not entirely reliable owing to the latency of motor impairment in comparison to sensory disturbance. Larger sample sizes would help to better correlate the response to USLIT in motor conduction disorders and other neuropathies, such as those affecting the common peroneal nerve.

In our study, all patients underwent surgical decompression—either ultrasound-guided or open—regardless of their response to USLIT. Follow-up data are currently being collected to identify correlations between USLIT and postoperative prognosis.

Follow-up with USG-NNNS is ongoing. Our findings are preliminary but promising. In patients with improved conduction velocity after USLIT, the postoperative results indicate a more successful outcome. This observation may prove important when attempting to provide a prognosis for nerve recovery in the event of planned decompression surgery.

## 5. Conclusions

We used quantitative data to show that USLIT can improve the neuropathic symptoms of TTS, with a correlative increase in conduction velocity. USLIT is a simple, reliable, and inexpensive objective test for the diagnosis of compressive neuropathy of the tibial nerve that could be applied for the diagnosis of disorders affecting other nerves.

The prognosis of surgical decompression could be more favorable in patients in whom nerve conduction improves after infiltration of lidocaine.

## Data availability statement

The raw data supporting the conclusions of this article will be made available by the authors, without undue reservation.

## Ethics statement

The studies involving human participants were reviewed and approved by Research Ethics Committee of Hospital Beata María. Code: US-PH-COT-2019. The patients/participants provided their written informed consent to participate in this study.

## Author contributions

ÁI, MV, and LV-Z designed the research study and performed the research. SB provided help and advice. ÁI analyzed the data and wrote the manuscript. All authors contributed to editorial changes in the manuscript and read and approved the final version of the manuscript.
